# PAS Kinase deficiency alters the glucokinase function and hepatic metabolism

**DOI:** 10.1038/s41598-018-29234-8

**Published:** 2018-07-23

**Authors:** A. Pérez-García, P. Dongil, V. Hurtado-Carneiro, E. Blazquez, C. Sanz, E. Alvarez

**Affiliations:** 1 Department of Biochemistry and Molecular Biology, Faculty of Medicine, Complutense University of Madrid, Institute of Medical Research at the Hospital Clínico San Carlos (IdISSC), Ciudad Universitaria, s/n, 28040 Madrid, Spain; 2 Spanish Biomedical Research Centre in Diabetes and Associated Metabolic Disorders (CIBERDEM), Madrid, Spain; 3 Department of Cell Biology, Faculty of Medicine, Complutense University of Madrid, Madrid, Spain

## Abstract

The liver controls metabolic homeostasis in response to fasting and refeeding periods. Glucokinase (GCK) adjusts hepatic glucose phosphorylation to blood glucose levels, acting as a glucose sensor. Our objective was to determine whether PAS kinase (PASK), a nutrient sensor, could be affecting the expression or activity of liver GCK and the response to fasting and refeeding states of key hepatic metabolic pathways. PASK-deficient mice have impaired insulin signaling (AKT overactivation). Furthermore, PASK deficiency modified the expression of several transcription factors involved in the adjustment to fasting and refeeding. *Foxo1* decreased under fasting conditions, while *Ppara* and *Pparg* were overexpressed in PASK-deficient mice. However, PEPCK protein levels were similar or higher, while the expression of *Cpt1a* decreased in PASK-deficient mice. By contrast, *Lxra* and *Chrebp* were overexpressed after refeeding, while the expression of *Acc* and *Fas* decreased in PASK-deficient mice. Likewise, with a decreased expression of *Gck* and increased nuclear location of the complex GCK-GCKR, GCK activity decreased in PASK-deficient mice. Therefore, PASK regulated some of the genes and proteins responsible for glucose sensing, such as glucokinase, and for insulin signalling, affecting glucose and lipid metabolism and consequently certain critical hepatic functions.

## Introduction

The liver plays a key role in metabolic homeostasis. It is the main site for the synthesis, metabolism, storage and redistribution of carbohydrates, proteins and lipids. It is especially critical in fasting/feeding responses. Thus, in the fed state, glycolytic products are used to synthesize fatty acids through *de novo* lipogenesis. By contrast, glycogenolysis and hepatic gluconeogenesis is the primary source of endogenous glucose production during fasting. Fasting duration could have major metabolic consequences, especially on the substrate used for hepatic glucose production. Aberrant metabolism in the liver therefore promotes insulin resistance, diabetes, and non-alcoholic fatty liver diseases.

Glucokinase (GCK, hexokinase type IV) is a critical enzyme controlling hepatic metabolism, regulating hepatic carbohydrate metabolism by acting as a glucose sensor. It triggers shifts in metabolism or cell function in response to changes in glucose levels, as occurs after a meal. It is expressed mainly in the liver^1^ and pancreatic β-cells^2^, as well as in neuroendocrine cells, jejunal enterocytes, and the brain^3–7^. In these cases, GCK is the enzyme that facilitates the phosphorylation of glucose to glucose 6-phosphate (G6P). The kinetic properties of GCK ensure that the rate of glucose phosphorylation is directly proportional to blood glucose levels, and also catalyzes the rate-limiting step of glucose catabolism. Accordingly, it is considered to be a true glucose sensor^2^, being involved in glucose-dependent insulin release by pancreatic β-cells.

Hepatic GCK acts in tandem with insulin in crucial functions in the liver, such as the maintenance of blood glucose and lipid homeostasis^8^, as well as glycogen synthesis and storage. Insulin also inhibits glycogenolysis and gluconeogenesis, and increases *de novo* lipogenesis. GCK expression and activity are regulated by transcriptional and posttranscriptional mechanisms. The hepatic GCK gene expression is insulin-dependent^9^, but it is also regulated posttranscriptionally through interaction with other proteins, highlighting the glucokinase regulatory protein (GCKR) that drives the subcellular location of GCK^10–12^.

The importance of GCK in maintaining glucose homeostasis is evidenced by the severe impacts that cause the mutations in the GCK gene. Thus, the loss of function of GCK in humans cause maturity-onset diabetes of the young type 2 (MODY2)^13^. By contrast, activating mutations generate persistent hyperinsulinemia^14^. Furthermore, hepatic GCK is also required for the proper activation of glycolytic and lipogenic gene expression in the liver^15^.

Insulin resistance in the liver contributes greatly to the development of type 2 diabetes mellitus^16–18^, and may also promote lipid synthesis, producing hepatic steatosis and further systemic insulin resistance^19^. This is due to lower hepatic insulin sensitivity that leads to postprandial hyperglycemia and increased hepatic glucose production, exacerbating hyperglycemia and chronic hyperinsulinemia in diabetics^20^.

PAS kinase (PASK) is a nutrient-sensing regulator of both glucose and energy metabolism homeostasis in mammals^21,22^. PASK-deficient mice are protected against obesity and the development of hepatic steatosis and the insulin resistance induced by a high-fat diet^23^.

Previous reports have advanced our understanding of the PASK function in certain aspects of glucose and lipid metabolism. However, there is no in-depth knowledge on the importance of PASK in the appropriate response to fasting/feeding states.

We have identified PASK in the hypothalamus (ventromedial and lateral areas), and its expression is regulated by fasting/refeeding conditions. Additionally, PASK-deficient mice record an altered nutrient response of the AMPK and mTOR pathways, not only in the hypothalamic areas involved in the control of food intake, but also in the liver^24^. The critical role of GCK as a glucose sensor in the liver and beta-cells prompted this research team to investigate the relationship and coordination between GCK and PASK functions in the liver, analyzing the response to fasted and refeeding states. In addition, PASK has been proposed as one of the possible targets for the treatment of the metabolic syndrome. We have also highlighted PASK’s potential role in the control of the key genes and proteins that lead to hepatic metabolic adaptation to fasting or feeding situations. We have focused accordingly on the insulin signaling pathway, gluconeogenesis, and fatty acid and triglyceride metabolism.

## Results

### Altered regulation of liver *Gck* and *Gckr* expression by feeding/fasting in PASK-deficient mice

**Figure 1:**
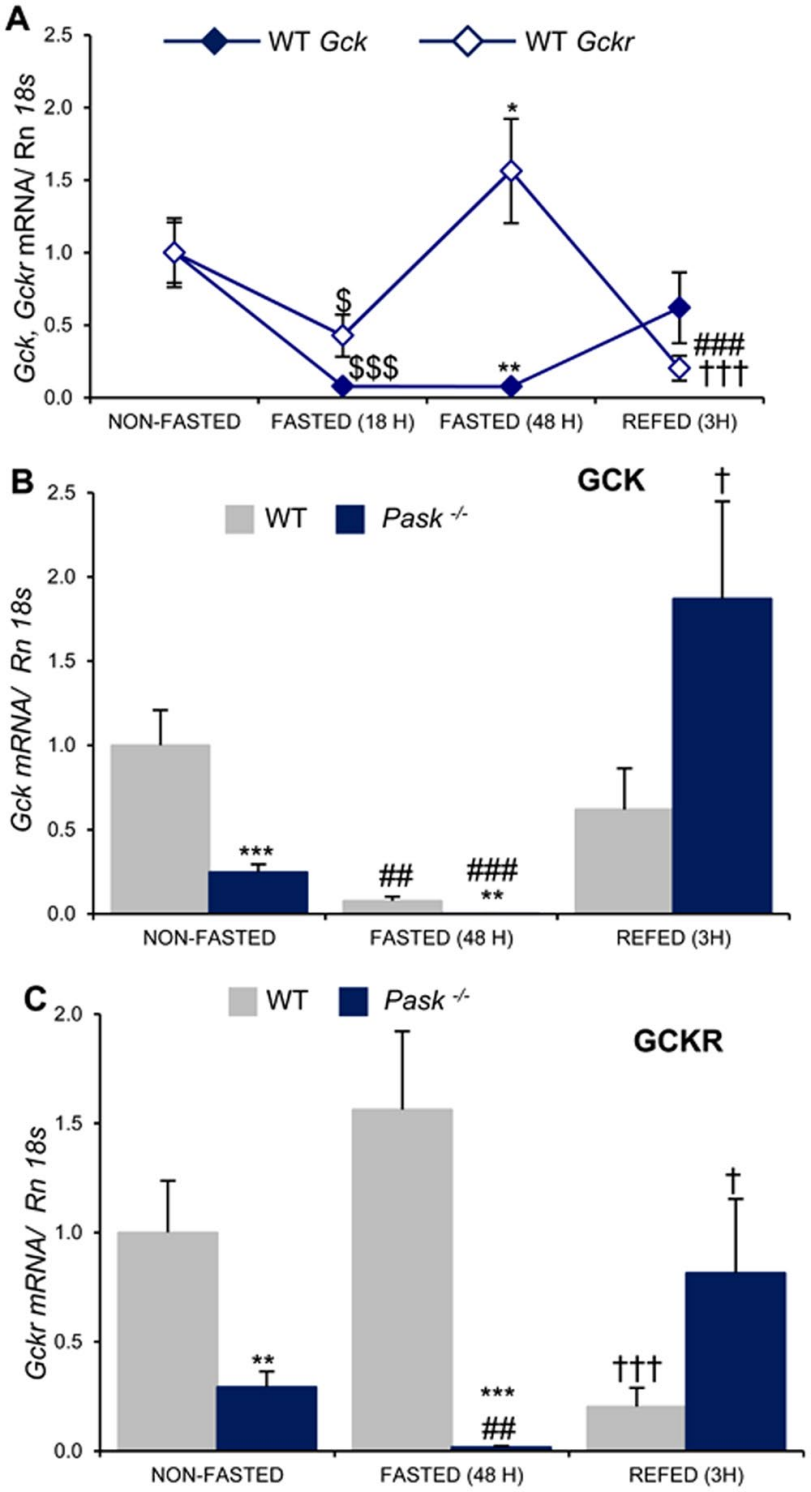
Effects of PASK deficiency on the response of hepatic *Gck* and Gckr mRNA levels to fasting/refeeding. Quantitative real-time PCR was used to analyze the expression of *Gck* and *Gckr* mRNA levels in liver from WT and PASK-deficient mice (*Pask*^−/−^). (**A**) The mRNA levels were measured in non-fasted (NON-FASTED), fasted (18 h, 48 h) (FASTED (18 H), FASTED (48 H)) and 3 h refeeding (REFED (3 H)) from WT mice, and normalized by *Rn18s*. The value obtained under non-fasted conditions was taken as 1. Results are means ± SEM; n = 4–5 animals per condition. The differences between groups were first tested with an ANOVA followed by pairwise t-test comparisons with the Tukey’s post hoc test in order to determine the differences between groups. ^$^*P* < 0.05; ^$$$^*P* < 0.001 non-fasted vs. fasted 18 h; *P < 0.05; **P < 0.01 non-fasted vs. fasted 48 h; ^###^*P* < 0.001 non-fasted vs. refeeding; and ^†††^*P* < 0.001 fasted 48 h vs. refeeding. (**B**) *Gck* mRNA from non-fasted (NON-FASTED), fasted 48 h (FASTED) and 3 h refeeding (REFED). **P < 0.01; ***P < 0.001 WT vs. *Pask*^−/−^; ^##^*P* < 0.01; ^###^*P* < 0.001 non-fasted vs. fasted 48 h; and †*P* < 0.05 fasted 48 h vs. refeeding. (**C**) *Gckr* mRNA from non-fasted (NON-FASTED), fasted 48 h (FASTED) and 3 h refeeding (REFED). **P < 0.01; ***P < 0.001 WT vs. *Pask*^−/−^; ^##^*P* < 0.01 non-fasted vs. fasted 48 h; and ^†^*P* < 0.05; ^†††^*P* < 0.001 fasted 48 h vs. refeeding.

*Gck* and *Gckr* expression was measured in the liver of WT and *Pask*^−/−^ mice under non-fasted, fasted (18 h or 48 h), and 3 h-refeeding conditions. Under fasting, the expression of both *Gck* and *Gckr* (Fig. 1A) was blocked in the WT. However, conversely to *Gck*, an increased expression of *Gckr* was observed in longer fasting periods (Fig. 1A).

Under normal (non-fasted) conditions, PASK-deficient mice recorded a ~3 times lower expression of *Gck* and *Gckr* than in the WT (Fig. 1B,C). Prolonged fasting blocked the expression of *Gck* and *Gckr* in *Pask*^−/−^ mice (Fig. 1B,C). By contrast, the expression of *Gck* and *Gckr* after refeeding recovered faster in the *Pask*^−/−^ mice than in the WT (Fig. 1B,C).

### Altered GCK and GCKR protein expression and sub-cellular location in PASK-deficient mice

**Figure 2:**
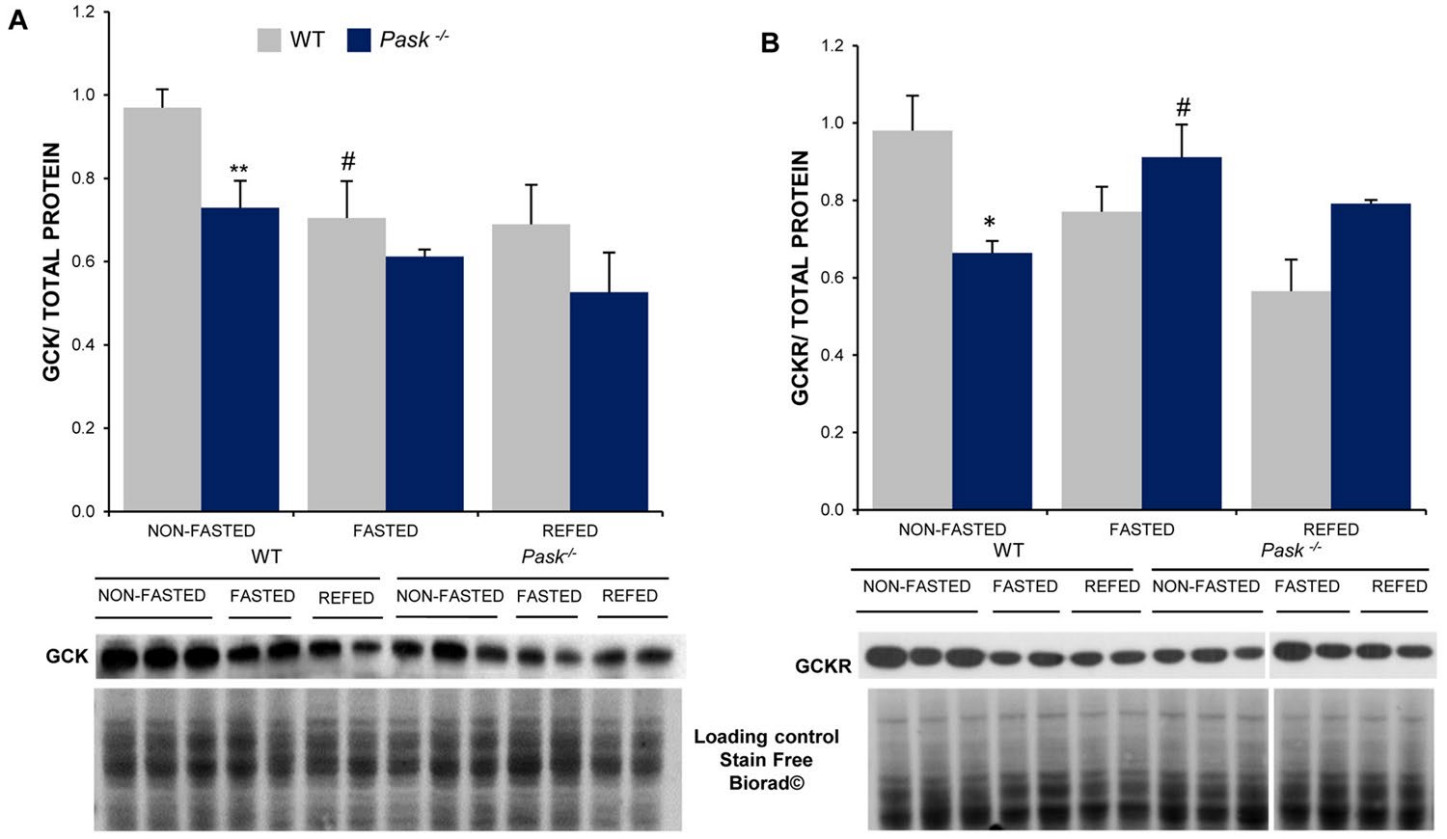
Effects of PASK deficiency on the expression of hepatic GCK and GCKR proteins in response to fasting/refeeding. Immunoblot analysis of GCK (**A**) and GCKR (**B**) in liver from WT and PASK-deficient mice (*Pask*^−/−^). Liver lysates from non-fasted (NON-FASTED), fasted 48 h (FASTED) and 3 h refeeding (REFED) were processed for western blot analysis. Bar graphs represent means ± SEM of the densitometric values normalized by total protein detected by Stain-Free (Total Protein SF) (Supplementary Fig. S1); n = 4–5 animals per condition. Representative western blots of the graphs and a fragment of same membranes with Stain-Free staining (total protein loading control) are also shown. The values were normalized by non-fasted WT mice. Tukey’s test was used to determine the differences between groups. *P < 0.05; **P < 0.01 WT vs. *Pask*^−/−^ and ^#^*P* < 0.05 non-fasted vs. fasted 48 h.

GCK and GCKR protein levels were also analyzed in the livers from the WT and *Pask*^−/−^ mice under non-fasted and fasted (48 h) and 3 h-refeeding conditions. The adjustment of GCK and GCKR proteins is reported to be much slower than changes in mRNAs levels, as both proteins have a low turnover ratio. GCK and GCKR protein expression was lower under the non-fasted condition in PASK-deficient mice (Fig. 2A,B). The effect of fasting was also observed at the level of GCK protein expression in the WT. However, fasting produced slight and opposite changes in protein levels, decrease in GCK and increase in GCKR in *Pask*^−/−^ mice. As expected, after refeeding the expressions of GCK and GCKR were similar to the fasted condition in both the WT and PASK-deficient mice (Fig. 2A,B).

**Figure 3:**
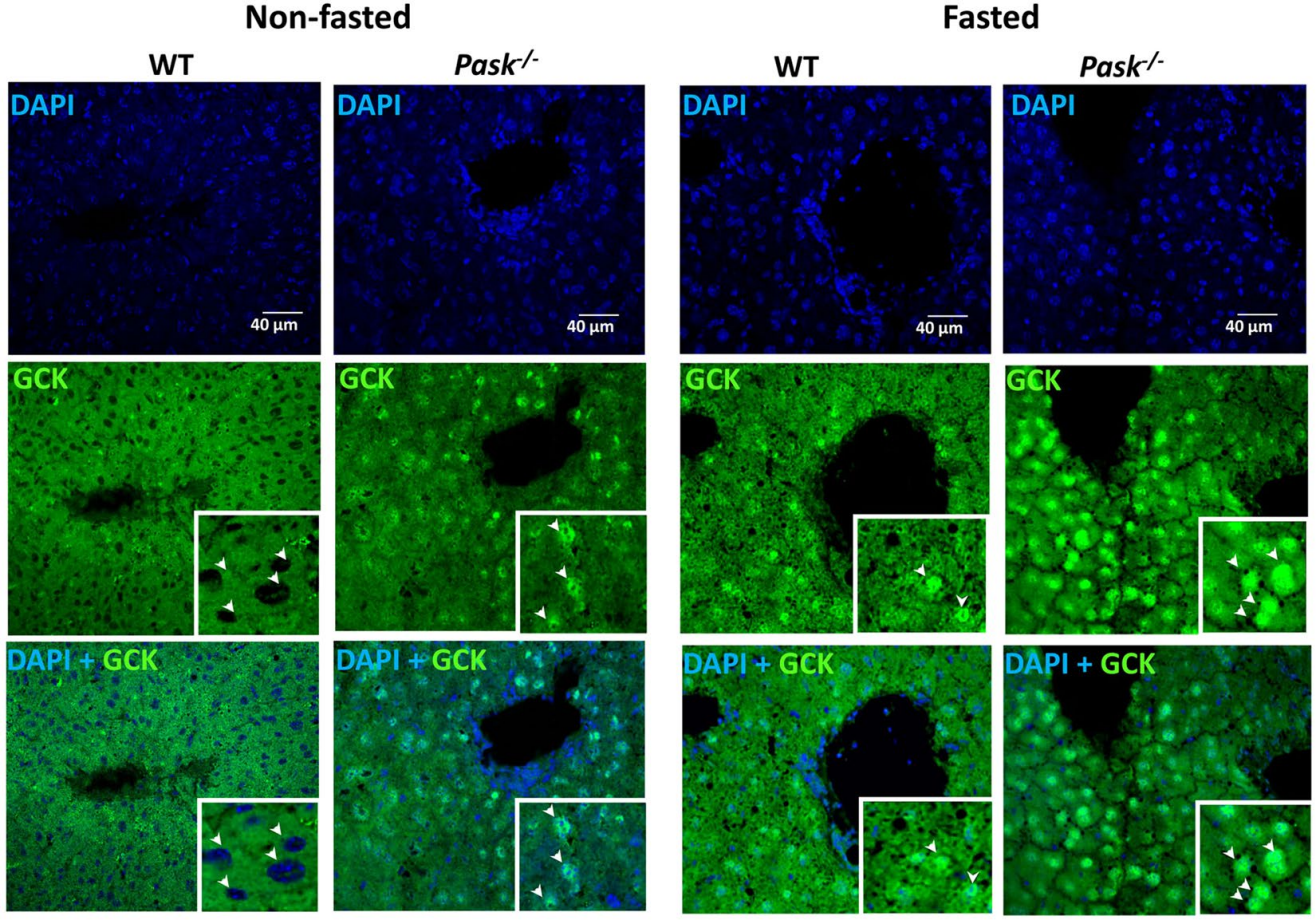
Effects of PASK deficiency on GCK sub-cellular location. Immunocytochemistry of GCK in the liver from non-fasted and fasted C57Bl/6 WT and *Pask*^−/−^ mice. GCK sub-cellular location was visualized in green (Alexa 488 coupled to a secondary antibody). Nuclei were stained with DAPI (blue fluorescence). Arrows indicate nuclei position. Inserts are a magnification to show details.

The sub-cellular location of GCK was also analyzed, as we knew that GCKR-GCK interaction drives the complex to nuclear localization, inhibiting GCK activity. The localization of GCK was examined by immunohistochemistry in liver sections from non-fasted and fasted WT and *Pask*^−/−^ mice. GCK was found mainly in the cytosol location in the hepatocytes from WT livers under non-fasting conditions. Nevertheless, GCK recorded a mainly nuclear location in *Pask*^−/−^ mice, with a similar pattern to that recorded by both types of mice under fasted conditions (Fig. 3).

### Enzymatic glucokinase activity falls in PASK-deficient mice

GCK activity among the WT and PASK-deficient mice was measured as glucose-phosphorylation activity under non-fasted conditions and as the resilience of GCK activity in the WT animals refed after prolonged fasting.

**Figure 4:**
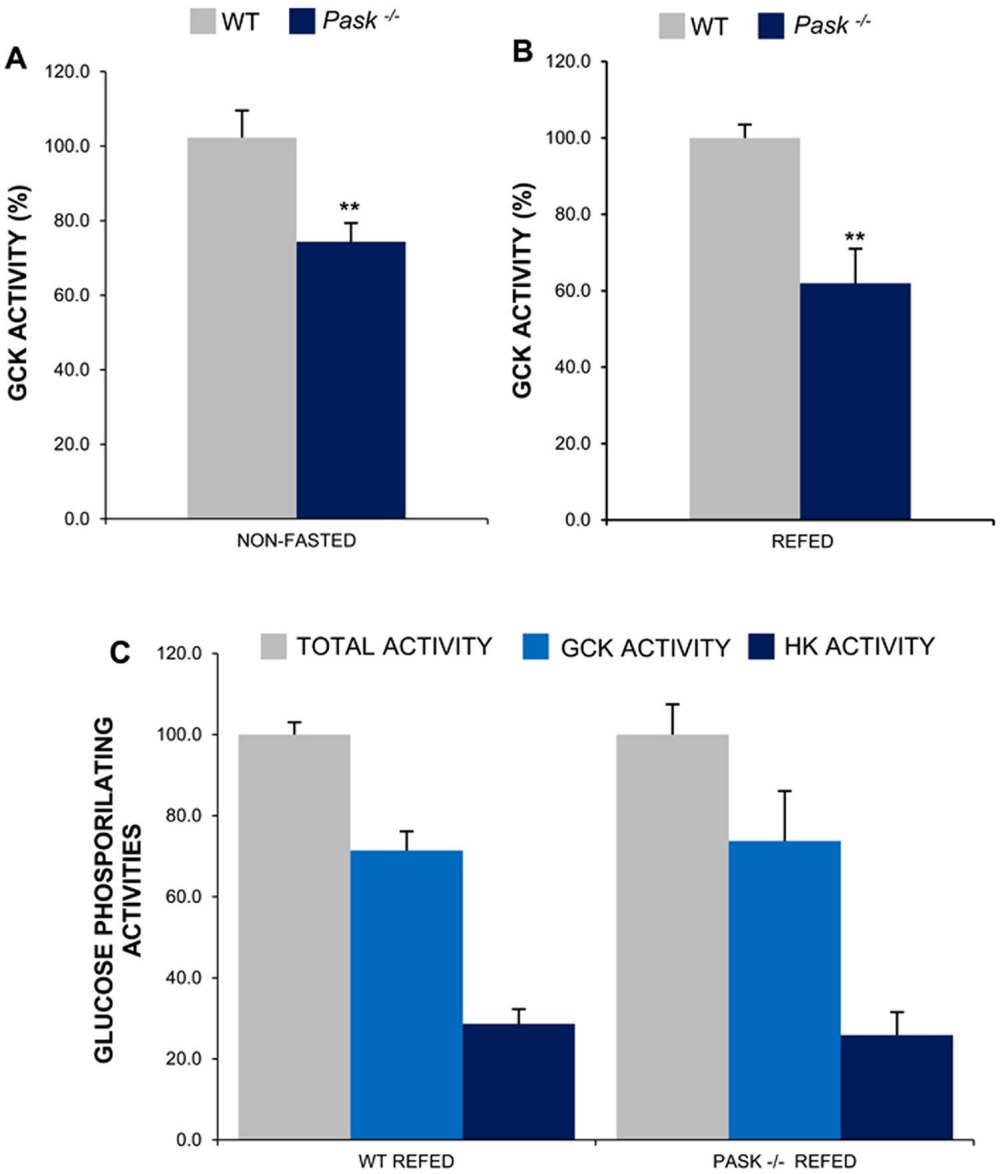
PASK-deficient mice have diminished glucokinase activity. Total glucose-phosphorylating activity assays were performed at 30 mM, whereas low-Km hexokinase (HK) activities at 0.3 mM glucose. GCK activity was obtained by the difference between individual values at high and low glucose. (**A**) Liver homogenates from non-fasted (NON-FASTED) liver from the WT and PASK-deficient mice (*Pask*^−/−^). (**B**) Liver homogenates from 3 h refeeding after fasting (REFED) from the WT and PASK-deficient mice (*Pask*^−/−^). GCK activity is given as means ± SEM as a percentage of the value of the control WT; n = 4–5 animals per condition. Tukey’s test was used to determine the differences between groups. **P < 0.01 WT vs. *Pask*^*−/−*^. (**C**) Low-Km hexokinase (HK) and glucokinase (GCK) activities are given as means ± SEM as a percentage of the total glucose-phosphorylating activity. The WT and PASK-deficient samples were both normalized to the total activity for each respective genotype.

Liver GCK activity was significantly lower in *Pask*^−/−^ mice under non-fasted conditions than in their WT controls (Fig. 4A). The recovery of hepatic GCK activity after fasting was ≈ 40% lower (p < 0.01) in refed *Pask*^−/−^ mice (Fig. 4B).

In order to rule out a different contribution by GCK or HK to the total phosphorylating activity in control or PASK-deficient mice, we analyzed the percentage of total glucose-phosphorylating activity that was due to GCK. The contribution of GCK to total glucose phosphorylating activity was similar at ~70% in both *Pask*^−/−^ and in the control WT (Fig. 4C).

### Impaired insulin signaling pathway in response to fasting or refeeding in PASK-deficient mice

Insulin signaling activates phosphatidylinositol-3,4,5-triphosphate kinase (PI3K) and leads to the further activation of AKT. Signaling through this pathway is also regulated by the phosphatase PTEN, which controls the phosphorylation state of PIP_3_, PI3K and AKT. We analyzed the expression and activation levels of AKT in livers from non-fasted, 48-h fasted, and after 3-h refeeding in the WT and *Pask*^−/−^ mice.

**Figure 5:**
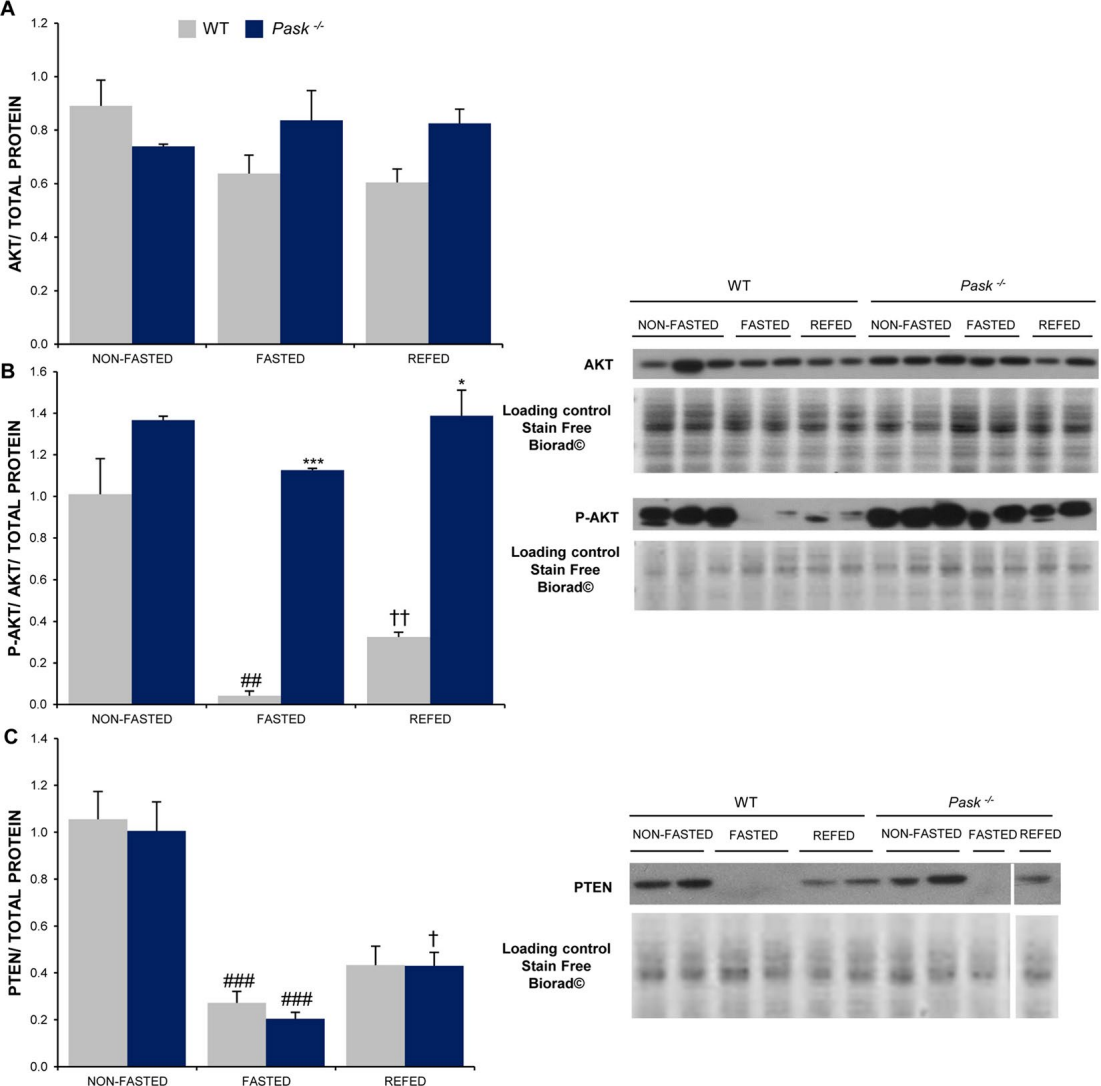
PASK-deficiency alters the insulin signaling pathway after fasting or refeeding states. Immunoblot analysis of phospho-AKT (Ser 473) (P-AKT) and total AKT (AKT) (**A**,**B**) and total PTEN (PTEN) (**C**) in the liver from the WT and PASK-deficient mice (*Pask*^−/−^). Liver lysates from non-fasted (NON-FASTED), fasted 48 h (FASTED) and 3 h refeeding (REFED) mice were processed for western blot analysis. Bar graphs represent the means ± SEM of the densitometric values normalized by total protein detected by Stain-Free Biorad**©** (Total Protein SF) (Supplementary Fig. S1); n = 4–5 animals per condition. Representative western blots of the graphs and a fragment of same membranes with Stain-Free Biorad**©** staining (total protein loading control) are also shown. The values were normalized by non-fasted WT mice. Tukey’s test was used to determine the differences between groups. *P < 0.05; ***P < 0.001 WT vs. *Pask*^−/−^; ^##^*P* < 0.01; ^###^*P* < 0.001 non-fasted vs. fasted 48 h; and ^†^*P* < 0.05; ^††^*P* < 0.01 fasted 48 h vs. refeeding.

PASK deficiency did not affect the total level of AKT, which under the conditions analyzed in *Pask*^−/−^ was similar to the WT (Fig. 5A). The activation state of AKT was detected with anti-phospho-AKT (Ser 473). The degree of AKT activation tends to be higher, although non-statistically significant, in *Pask*^−/−^ under non-fasted conditions compared to their WT controls. However, while fasting sharply reduced AKT activity in the WT, AKT activation remained high in *Pask*^−/−^ mice, regardless of the feeding or fasting conditions (Fig. 5B).

We tested the levels of hepatic PTEN in *Pask*^−/−^ mice, finding that the PTEN protein level was lower in the fasted state and begins to increase after refeeding and the expression level was higher under non-fasted conditions in both *Pask*^−/−^ and the WT (Fig. 5C).

### Altered response of hepatic metabolic genes to fasting and refeeding in PASK-deficient mice

**Figure 6:**
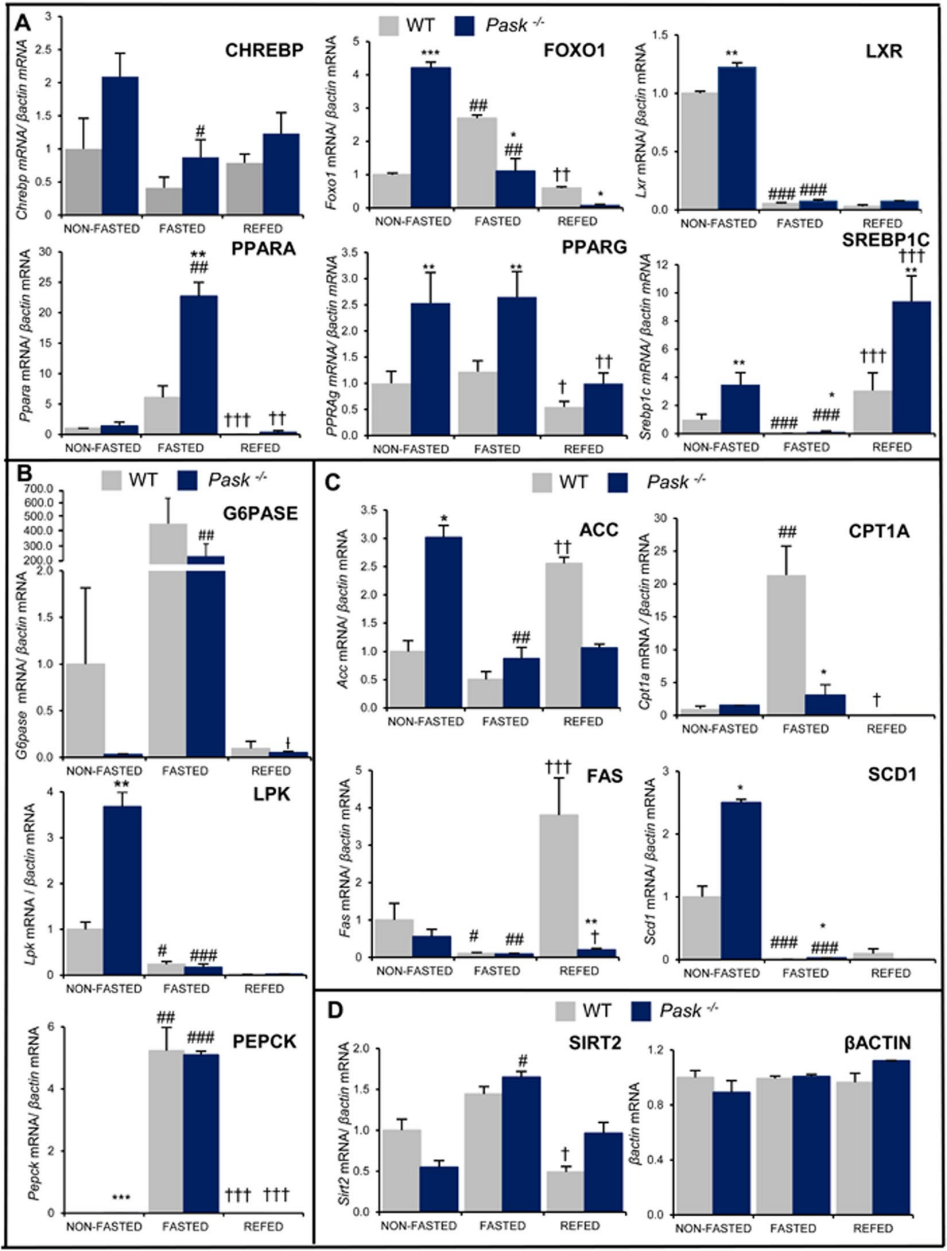
Effects of PASK deficiency on the expression of several hepatic genes. Quantitative real-time PCR was used to analyze the expression of several genes as shown (Table 2). The mRNA levels were measured in non-fasted (NON-FASTED), fasted 48 h (FASTED) and 3 h refeeding (REFED) WT and PASK-deficient mice (*Pask*^−/−^). The mRNA levels of the different genes were normalized by mRNA of *β-actin* used as housekeeping gene. The value obtained in the non-fasted WT mice was taken as 1. Results are means ± SEM; n = 4–5 animals per condition. Tukey’s test was used to determine the differences between groups. *P < 0.05; **P < 0.01; ***P < 0.001 WT vs. *Pask*^−/−^; ^#^*P* < 0.05; ^##^*P* < 0.01; ^###^*P* < 0.001 non-fasted vs. fasted 48 h; and ^†^*P* < 0.05; ^††^*P* < 0.01; ^†††^*P* < 0.001 fasted 48 h vs. refeeding.

We evaluated the role of PASK in the hepatic function by analyzing the expression of the transcription factors and genes that regulate the main metabolic pathways in the liver, such as gluconeogenesis, fatty acid, and triglyceride metabolism (Fig. 6). mRNA levels were measured by real-time RT-PCR in the liver for non-fasted or 48-h fasted conditions and after 3 h-refeeding in the WT and *Pask*^−/−^ mice.

Fasting increased the expression of hepatic: *Foxo1, Ppara, G6pase, Pepck, Cpt1a, Sirt2* in the control WT (Fig. 6). The increased expression of these genes is consistent with the activation of gluconeogenesis and the increased transport of fatty acids into mitochondria. However, PASK-deficient mice recorded a decrease in the expression levels of genes induced by fasting in the WT: *Foxo1, Cpt1a, G6pase*, and an increased expression of *Ppara* and *Pparg*. Refeeding the control WT quickly increased (3 h) the expression of *Srebp1c, Acc* and *Fas* compared to fasted WT mice. However, other genes needed sustained feeding (non-fasted state) to increase the expression, such as *Lxra, Chrebp, Lpk* and *Scd1*. These data confirm that physiological refeeding immediately blocks the expression of the genes stimulated by fasting (*Foxo1, Ppara*, *Pepck, G6pase, Cpt1a, Sirt2*), inhibiting the expression of gluconeogenic and *Cpt1a* enzymes. Moreover, feeding stimulates the level of expression of *Srebp1c*, as occurred with *Gck*, inducing glycolytic and lipogenic genes in the control WT. Nevertheless, PASK-deficiency disrupted *Acc* and *Fas* expression, and so *de novo* lipogenesis could be impaired. However, *Lxra* and *Srebp1c* expression was significantly higher, and the level of expression of *Chrebp* and *Sirt2* increased slightly after refeeding in *Pask*^−/−^ mice respect to the WT. Finally, altered gene expression was also observed in non-fasted liver from PASK-deficient mice compared to the WT. For example, there was a significantly higher expression of mRNA coding to *Foxo1, Lxra*, *Pparg, Srebp1c, Acc, Lpk, Pepck* and *Scd1*.

### Altered PEPCK and SREBP1 protein expression in PASK-deficient mice

**Figure 7:**
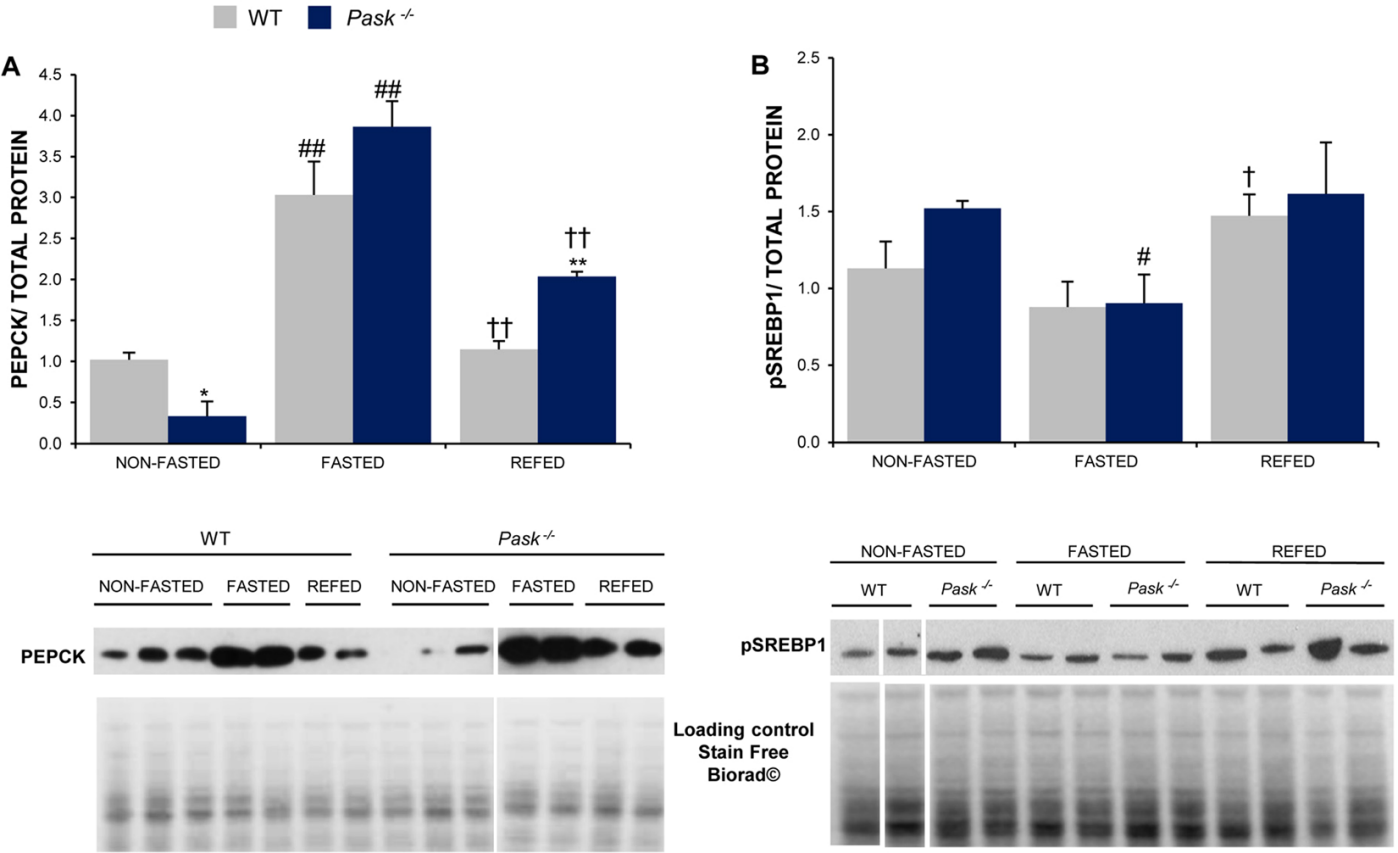
PASK-deficiency alters PEPCK and SREBP1 protein expression. Immunoblot analysis of PEPCK (PEPCK) (**A**) and the level of precursor (pSREBP1) (**B**) in the liver from the WT and PASK-deficient mice (Pask^−/−^). Liver lysates from non-fasted (NON-FASTED), fasted 48 h (FASTED) and 3 h refeeding (REFED) mice were processed for western blot analysis. Bar graphs in (**A**,**B**) represent means ± SEM of the densitometric values normalized by total protein detected by Stain-Free (Total Protein SF) (Supplementary Fig. S1); n = 4-5 animals per condition. Representative western blots of the graphs and a fragment of same membranes with loading control Stain Free Biorad**©** staining (total protein loading control) are also shown. The values were normalized for the non-fasted WT mice. Tukey’s test was used to determine the differences between groups. *P < 0.05; **P < 0.01 WT vs. Pask^−/−^; ^#^P < 0.05; ^##^P < 0.01 non-fasted vs. fasted 48 h; and ^†^P < 0.05; ^††^P < 0.01 fasted 48 h.

The levels of gluconeogenic PEPCK protein and membrane SREBP1 precursor protein (pSREBP1) were also analyzed in the liver from the WT and *Pask*^−/−^ mice under non-fasted and 48-h fasting conditions and after 3h-refeeding (Fig. 7A,B). Fasting increased the expression of hepatic PEPCK protein in both the control WT and PASK-deficient mice. The expression level of PEPCK decreased after refeeding in both types of mice, although the reduction was slower in *Pask*^−/−^ mice. Under the non-fasted condition, however, the expression level was lower in the PASK-deficient mice than in the control WT (Fig. 7A). pSREBP1 analyzed in cytoplasmic fraction increased in refeeding compared to fasting conditions in both the control WT and PASK-deficient mice (Fig. 7B).

## Discussion

The liver has a crucial role to play in maintaining glucose and lipid homeostasis in both fasted and postprandial states. Insulin prompts changes in liver metabolism that allow adapting to daily periods of fasting and refeeding. The release of insulin in response to high levels of glucose inhibits gluconeogenesis and activates glycolysis and lipogenesis. These metabolic actions are controlled by the expression and activation/inhibition of key proteins, some of which have been studied here.

PASK has been involved in glucose and energy metabolism homeostasis^21–23^. PASK-deficient mice are reportedly resistant to diet-induced obesity^23^. Nevertheless, a lower expression of PASK has been reported in pancreatic islets from type 2 diabetic patients^25^. We have previously described how PASK acts as a nutrient and hormonal sensor in the hypothalamic areas involved in feeding behavior^24,26^. The expression of PASK in this organ is regulated by states of fasting/refeeding in the hypothalamic areas involved in food intake regulation. Furthermore, *Pask*^−/−^ mice have recorded an impaired response of AMPK and mTOR/S6K1 pathways to fasting/refeeding conditions in those hypothalamic areas, as well as in the liver^24^. Thus, our previous data indicate that PASK deficiency leads to an over-activated AMPK, under conditions in which this sensor might remain inactive (refeeding). These routes also have a key role to play in the liver’s adaptive changes to states of fasting and refeeding. Thus, the activation of AMPK in liver and muscle decreases circulating glucose and lipid levels^27^, and inhibits PI3K/AKT signaling^28^. Broadly speaking, AMPK activation switches on catabolic pathways and inhibits anabolic ones. Although there are many studies involving AMPK and other sensors in the adaptation of liver to fasting and refeeding situations, little or nothing is known about the role of PASK in this key hepatic function. This research has therefore investigated how PASK deficiency affects liver responses to fasting and refeeding. Our data show that *Pask*^−/−^ mice have higher levels of activated AKT in the liver under both fasted and refeeding conditions. These results correlate with the slight overexpression of S*irt2* gene in PASK-deficient mice, mainly under refeeding conditions. In the light of previous results, the overexpression of SIRT2 would promote AKT activation^29^. It has been reported that AKT mediates insulin action on glucose and lipid homeostasis^30^. The activation of this pathway inhibits gluconeogenic gene expression, and activates glycolysis and lipogenesis. During fasting, FoxO transcription factors induce the expression of genes that promote hepatic glucose production (*Pepck*, *G6Pase*)^31^, and decrease the expression of *Gck*^32,33^.

During fasting, furthermore, glycogenolysis and hepatic gluconeogenesis are activated, maintaining blood glucose homeostasis. Glycolysis is shut down, and fatty acids are used as an energy source. Our results show that prolonged fasting increases the expression of hepatic transcription factors and metabolic genes that should promote the activation of gluconeogenesis and the genes involved in the transport of fatty acids into mitochondria in WT mice. However, PASK-deficient mice recorded changes in the level of expression of several genes induced by fasting, decreasing *Foxo1* and *Cpt1a* and increasing *Ppara* and *Pparg*. These data might also respond to AKT overactivation that may phosphorylate and inhibit FoxO1^34^. However, the expression of cytosolic PEPCK protein that supported hepatic gluconeogenesis from glycogenic amino acids increased in fasted PASK-deficient mice, and the level of PEPCK remained higher than in the control WT mice after refeeding. A possible explanation for these results may also involve the acetylation/deacetylation mechanism that regulates PEPCK protein stability^35^. We have therefore checked the expression of the mRNA coding to SIRT2, the protein that deacetylates PEPCK. Our data show that *Sirt2* expression was slightly higher in fasted PASK-deficient mice, and it also remains higher when they are refed. These data suggest that the deacetylation of PEPCK could increase its stability and regulate PEPCK protein expression. These data are difficult to interpret because protein activity often depends on regulatory mechanisms that control transcription, post-translational modifications that might also regulate stability and the half-life of the protein, or sometimes their subcellular localization. Previous reports on rodents and humans have not found a direct relationship between type 2 diabetes and the altered expression of PEPCK or G6Pase^36,37^. The indirect regulation of hepatic glucose production cannot be excluded by insulin actions in other organs^38,39^. Regarding the lower expression of *Cpt1a* under fasting conditions in PASK-deficient mice, we know that they do not show symptoms of CPT1A deficiency^40^. Since blood glucose levels do not change significantly under 24 h or 48 h fasting in WT and PASK-deficient mice or even glucose circulating levels were slightly higher in PASK-deficient mice^24^.

Likewise, our data show that prolonged fasting decreased *Gck* while increasing *Gckr* expression in the WT mice, in accordance with previous reports^8,41^. By contrast, fasting blocked the expression of both *Gck* and *Gckr* genes in PASK-deficient mice. However, the recovery of the expression of *Gck* and *Gckr* was faster after refeeding in PASK-deficient mice than in the control WT. GCK is the key rate-limiting enzyme of hepatic glycolysis, enhancing the flow of glucose via glycolysis, and increasing acetyl-CoA production for lipogenesis. Liver GCK expression is regulated at transcriptional level mainly by insulin, and repressed by glucagon^8^. The insulin stimulatory effect is reportedly mediated by hepatocyte nuclear factor-4α (HNF4a)^42^. It has been also reported that FoxO1 might interact with HNF4a, decreasing the GCK expression^33,43^. GCK promoter contains additional binding sites for other transcription factors such as SREBP1c^44^ and also responds to PPARg, hypoxia and metabolic stress. The increased expression of GCK in refeeding states is consistent with the overactivation of AKT after refeeding in PASK-deficient mice. These data might explain the more rapid increase in *Gck* and *Gckr* mRNA levels after refeeding in PASK-deficient mice. This effect was quickly reversed consistent with AKT activity, which at the basal condition was similar in the *Pask*^−/−^ and control WT mice. Thus, non-fasted PASK-deficient mice recorded a decrease in both *Gck* and *Gckr* gene expression corresponding to a slight reduction in GCK and GCKR protein expression. These results also fit the data for GCK activity, which was lower in *Pask*^−/−^ mice. Defects in the expression level of GCK may contribute to the dysregulation of glucose metabolism in type 2 diabetes.

GCK activity is also dependent on subcellular location. The GCKR-GCK complex is located in the nucleus. Elevated glucose concentrations lead to GCK release from the complex and its translocation to the cytoplasm where it acts^45,46^. There are also cytosolic GCK binding proteins, such as the bifunctional protein (PFK2/FBPase2) responsible for synthesizing and degrading fructose 2, 6 bisphosphate, phosphofructo-2-kinase fructose 2, 6 bisphosphatase-2^47^. This protein is modified by phosphorylation/dephosphorylation, altering its capacity to interact with GCK, and therefore its activity. Although we have not studied how PFK2/FBPase2 is regulated by PASK deficiency, there is evidence to show that the rate of glucose phosphorylation by GCK is inversely related to the activity of AMPK, which phosphorylates PFK2/FBPase2 and GCKR^12^. Our previous report shows that hepatic AMPK activity increased in refed PASK-deficient mice compared to the WT^24^, possibly contributing to the lower hepatic GCK activity found in *Pask*^−/−^ mice. Furthermore, our current results support the impaired GCK activity in PASK-deficient mice, as it has a mainly nuclear location under non-fasted conditions. The lowest GCK activity may also depend on the inhibition of *de novo* lipogenesis in these mice, as one of the key functions of GCK is to stimulate glycolysis and provide substrates for lipogenesis. It has been reported that the expression of lipogenic genes is impaired in PASK-deficient mice. The metabolic changes after feeding are mediated mainly by insulin. Its effect in lipogenesis is dependent on both the transcriptional expression of *Srebp1* gene^48–50^ and the proteolytic activation of SREBP1c precursor^51^. Previous results show that SREBP1 activation by proteolytic maturation is PASK-dependent^52^. Our data confirm that the hepatic expression of lipogenic genes induced after refeeding, such as *Acc*, *Fas* and *Scd1*, was absent in *Pask*^−/−^ mice. Nevertheless, we found that mRNA coding to SREBP1c was overexpressed under all the conditions studied in PASK-deficient mice, as opposed to a previously reported decrease^52^. However, measuring the protein level confirmed that the expression of the SREBP precursor was similar in PASK-deficient and control WT mice. Therefore, the data on the protein and mRNA of SREBP1c do not correlate. Our results show that after refeeding, insulin levels were higher, AKT was overstimulated, and *Lxra* and *Pparg* gene expression was higher in *Pask*^−/−^ than in the WT mice. All these conditions have already been reported as potent activators of the transcription of *Srebp1c* gene^50,53^. We observed a slight increase in *Fas* and a higher expression of *Acc* and *Scd1* mRNA under non-fasted conditions. Previous reports indicate that the deletion of *Srebp1c* gene in mice decreases fatty acid synthesis by 50%. The treatment of those mice with an LXR agonist leads to the recovery of a fraction of lipogenic gene expression and an increase in fatty acid synthesis^54^. A further hypothesis is that *Chrebp* gene overexpression after refeeding in PASK-deficient mice could regulate the expression of *Lpk*, *Acc* and *Scd1*, as previously reported^55^. However, it must be taken into account that the synthesis of malonyl-CoA by ACC1 could be compensated by ACC2 as described in liver-specific ACC1-deficient mice^56^. Our data also suggest that SREBP1c could limit, but is not critical for *Gck* expression in the liver of refed *Pask*^−/−^ mice, in accordance with previous reports^54,57^.

The GCK expression has been related to obesity. Thus, decreased GCK activity^58,59^ and reduced expression have been reported in diabetic patients with HbA1c > 7.0^37^. The low expression and activity of GCK in PASK-deficient mice could be an indicator of early symptoms of the development of type 2 diabetes, although no evidence was found using a glucose and insulin tolerance test^23,60^, and also glucose circulating levels were similar^24^. We suggest that one explanation is the absence of *de novo* lipogenesis in these mice, but additional studies are needed to support this hypothesis.

Our data indicate that PASK deficiency alters the hepatic response to fasting, although PEPCK protein levels were similar or higher than in WT mice. Similarly, although the expression of *Cpt1a* decreased in fasted PASK-deficient mice no hypoglycemia were observed after fasting (24 h or 48 h) in PASK-deficient mice. Likewise, GCK activity was reduced in *Pask*^−/−^ mice for two reasons: on the one hand, the lower protein expression, and on the other, its mainly nuclear location. We cannot rule out the fact that this effect may be partly due to the blocking of lipogenesis that characterizes PASK-deficient mice. In addition, the conversion of excess carbohydrate to lipids might also be limited by the low levels of ACC and FAS, although the gene coding to ACC and LPK was overexpressed under non-fasted conditions.

## Material and Methods

### Experimental Animals

All the procedures involving animals were approved by approved by the animal welfare committee of Madrid Complutense University (DC 86/609/EU), and met the guidelines for the care of animals specified by the European Community. The animals used were 10- to 16-week-old males (25–30 g), C57Bl/6 wild type (WT) and PASK-deficient mice back-crossed into C57Bl/6 for at least 12 generations^61^. The animals were fed *ad libitum* with a standard pellet diet, and housed at a constant temperature (21 °C) on a 12 h light-dark cycle, with lights on at 8 a.m.

### Animal treatment and liver extraction

*Pask*^−/−^ and WT mice were maintained in standard feeding conditions (ad libitum) (non-fasting), and then fasted for 48 hours. After the fasting period, some specimens were refed for three hours. The mice were then decapitated, and their liver was removed and immediately frozen in liquid nitrogen prior to use.

### Immunohistochemistry

A liver sample was fixed in 4% (w/v) paraformaldehyde, cryoprotected in 30% sucrose, frozen and sectioned at 20 µm. Glucokinase detection was performed as previously described^62^. Briefly, the sections were washed with PBS, permeabilized for 20 min with PBS-0.4% (v/v) Triton X-100, and blocked for one hour by incubation with (PBS, 10% (v/v) goat serum, 0.1% (v/v) Triton X-100). The liver sections were then incubated with an anti-GCK antibody (Table 1), diluted 1:100 in a blocking solution. For fluorescence detection, sections were incubated with Alexa Fluor® 488 coupled to an anti-rabbit secondary antibody (GeneTex, Inc., San Antonio, CA, USA), diluted 1:200. The nucleus DNA was stained by adding 1 µg/ml of 4,6-diamidino-2-phenylindole (DAPI) in PBS. The images were taken with a Leica TCS SP2 laser scanning spectral confocal microscope mounted on an inverted DM IRE 2 (Leica Microsystems, Wetzlar, Germany). Confocal fluorescence images were analyzed using LCS Lite software, also from Leica.

**Table 1:** Antibodies and conditions used for western blot assays.

Antibody	Host	Manufacturer	Dilution used
Anti-GCK	Rabbit	Santa Cruz Biotechnology, California, USA	1:1000
Anti-GCKR	Rabbit	Santa Cruz Biotechnology, California, USA	1:1000
Anti-AKT	Rabbit	Cell Signaling, Danvers, MA, USA	1:1000
Anti-phospho AKT1/PKBα (Ser 473)	Mouse	Milipore Iberica, Madrid, Spain	1:1000
Anti-PTEN	Rabbit	Milipore Iberica, Madrid, Spain	1:500
Anti-PEPCK	Rabbit	Santa Cruz Biotechnology, California, USA	1:1000
Anti-SREBP1	Rabbit	Santa Cruz Biotechnology, California, USA	1:1000
Anti-Rabbit-HRP	Goat	Milipore Iberica, Madrid, Spain	1:5000
Anti-Mouse-HRP	Goat	Bethyl Laboratories, Montgomery, USA	1:5000

### Real-time polymerase chain reaction

Total RNA was extracted from the livers with TRIzol (Life Technologies, Barcelona, Spain). cDNA synthesis was developed using the High-Capacity cDNA Archive Transcription Kit (Applied Biosystems), using 2 µg of RNA as template, following the manufacturer’s instructions. The template used consisted of four microlitres of a 1:10 dilution of the cDNA. Either TaqMan® Assay (Applied Biosystems, Foster City, CA) or SYBR Green® Assay (Applied Biosystems) was used to quantify the mRNA levels by real-time quantitative RT-PCR in a 7900HT Fast Real-Time PCR System (Applied Biosystems). The details of the primers and probes are provided in Table 2. *18s* and *β-actin* housekeeping genes were used for normalization. In the case of SYBR Green Assay, a standard curve was previously generated in each real-time PCR assay, involving tenfold serial dilutions of the cDNA samples.

**Table 2:** Identification of primers used in the different gene expression assays.

Gene	Mouse Forward primer	Mouse Reverse primer
Acc (ACC)	5′-CCTCTTCCTGACAAACGAG-3′	5′-TCCATACGCCTGAAACATG-3′
Actb (β-ACTIN)	5′-CTCTCTTCCAGCCTTCCTTC-3′	5′-GGTCTTTACGGATGTCAACG-3′
Cpt1a (CPT1A) ChREBP (CHREBP)	5′-CATGTCAAGCCAGACGAAG-3′ 5′ -CTGGGGACCTAAACAGGAGC-3′	5′-TGGTAGGAGAGCAGCACCT-3′ 5′ -GAAGCCACCCTATAGCTCCC-3′
Fasn (FASN)	5′-AAGGCTGGGCTCTATGGATT-3′	5′-GGAGTGAGGCTGGGTTGATA-3′
Foxo1 (FOXO1)	5′-GACAGCCGCGCAAGACCAG-3′	5′-TGAATTCTTCCAGCCCGCC-3′
G6pase(G6PASE)	5′-TTACCAAGACTCCCAGGACTG-3′	5′-GAGCTGTTGCTGTAGTAGTCG-3′
L-pk (L-PK)	5′-TTGCTCTACCGTGAGCCTC-3′	5′-ACCACAATCACCAGATCAC-3′
Lxra (LXRA)	5′-GCCCTGCACGCCTACGT-3′	5′-TAGCATCCGTGGGAACATCA-3′
Pepck (PEPCK)	5′-CCACAGCTGCAGAACA-3′	5′-GAAGGGTCGCATGGCAAA-3′
Ppar-α (PPARα)	5′-TGTTTGTGGCTGCTATAATTT-3′	5′-GCAACTTCTCAATGTAGCCTA-3′
Ppar-γ(PPARγ)	5′-GTGCCAGTTTGCATCCGTAGA-3′	5′-GGCCAGCATCGTGTAGATGA-3′
Scd1 (SCD1) Sirt2 (SIRT2)	5′-CTGACCTGAAAGCCCGAAG-3′ 5′-AGCCAACCATCTGCCACTAC-3′	5′-GCGTTGAGCACCAGAGTGTA-3′ 5′-CCAGCCCATCGTGTATTCTT-3′
Srebp-1c(SREBP1C)	5′-GGAGCCATGGATTGCACTT-3′	5′-GCTTCCAGAGAGGAGGCCAG-3′

Real-time polymerase chain reaction (sybr green qpcr).

### Liver protein detection by western blot

A tiny piece of frozen liver (~150 mg) was immediately lysed in a RIPA buffer (PBS, 1% NP-40, 0.5% sodium deoxycholate, 1 mM PMSF, 10 mM Leupeptin, 1 mM Na_2_VO_4_, 25 mM Na_4_P_2_O_7_, 10 mM FNa) and a protease inhibitor cocktail (Roche Diagnostics, Mannheim, Germany). The tissues were then immediately exposed to microwave irradiation for 5 s, and then homogenized as previously described^26^. Total and activated forms of proteins were detected by SDS-PAGE western blotting using the antibodies described in Table 1, followed by incubation with the specific secondary antibodies bound to HRP. Finally, chemiluminescence was recorded and quantified using Quantity One software (Bio-Rad, GS-800 Densitometer). The normalization by housekeeping protein was impossible since its expression was different in PASK-deficient mice or changed with the experimental conditions used. Then, Stain-Free staining was used as loading control^63,64^ alternative to Ponceau or other, since Stain-Free Technology outperform Ponceau staining and are more consistent than housekeeping proteins as a loading control^65^. The detection of proteins on Stain-Free gels is based in modification of tryptophan residues from proteins using a trihalo compound. After the transference to a membrane the modified tryptophans give a fluorescent signal by illumination with UV light, proportional to protein load, that can be readily detected and quantified to use as loading control.

### Assay of glucokinase phosphorylating activities

Glucose-phosphorylating activities were measured using a spectrophotometric assay (Roncero *et al*.^6^). The activity analysis involved assays at two glucose concentrations: 0.3 mM for low-Km hexokinase (HK) activities (a concentration at which GK is essentially inactive) and 30 mM glucose (a concentration at which all phosphotransferase activities were measured). The enzyme reaction was performed using 40 μg of total protein extract in 200 μl of a reaction solution consisting of 100 mM Tris-HCl (pH 7.4), 150 mM KCl, 0.1% BSA, 1 mM MgCl_2_, 5 mM ATP-MgCl_2_, 2 mM NADP^+^ and 5.5 units of G6PDH from *Leuconostoc mesenteroides*. NADH production was measured by recording the fluorescence emitted in a spectrophotometer (Varioskan, Thermo Fisher Scientific). The fluorescence values were extrapolated from a standard curve plotted with dilutions of known concentrations of NADPH. GCK activity was calculated as the difference between phosphorylating activity at high and low glucose.

### Statistical analyses

Data are presented as means ± SEM. For experiments with multiple comparisons, differences between groups were first tested with an ANOVA, followed by pairwise t-test comparisons with Tukey’s post hoc test to determine the differences among groups. Data were considered statistically significant at P ≤ 0.05. Statistical analyses were performed using GraphPad Prism software.

## Electronic supplementary material

Supplementary Information
