# Supplementary material for: PAS Kinase deficiency alters the glucokinase function and hepatic metabolism
Source: Sci Rep. 2018 Jul 23;8:11091. doi: 10.1038/s41598-018-29234-8 (PMC6056484; doi:10.1038/s41598-018-29234-8)
Supplement: Supplementary file 1 — Supplementary Information [file 41598_2018_29234_MOESM1_ESM.pdf]

PAS Kinase deficiency alters the glucokinase function and hepatic metabolism

Pérez-García A[1,3], Dongil P[1,3], Hurtado-Carneiro V[1,2], Blázquez E[1,2], Sanz C[2,3# *] and Álvarez E[1,2#]

[#] These authors have contributed equally to this work

[1]Department of Biochemistry and Molecular Biology, Faculty of Medicine, Complutense University of Madrid, Institute of Medical Research at the Hospital Clínico San Carlos (IdISSC), Ciudad Universitaria, s/n, 28040 Madrid, Spain. [2]Spanish Biomedical Research Centre in Diabetes and Associated Metabolic Disorders (CIBERDEM), Spain, URL www.ciberdem.org. [3]Department of Cell Biology, Faculty of Medicine, Complutense University of Madrid, Spain

* Corresponding autor (C. Sanz): Departamento de Biología Celular, Facultad de Medicina, Universidad Complutense, Plaza S. Ramón y Cajal, s/n, 28040-Madrid, Spain. Fax. +34 913 941 691, e-mail: mcsanz@med.ucm.es

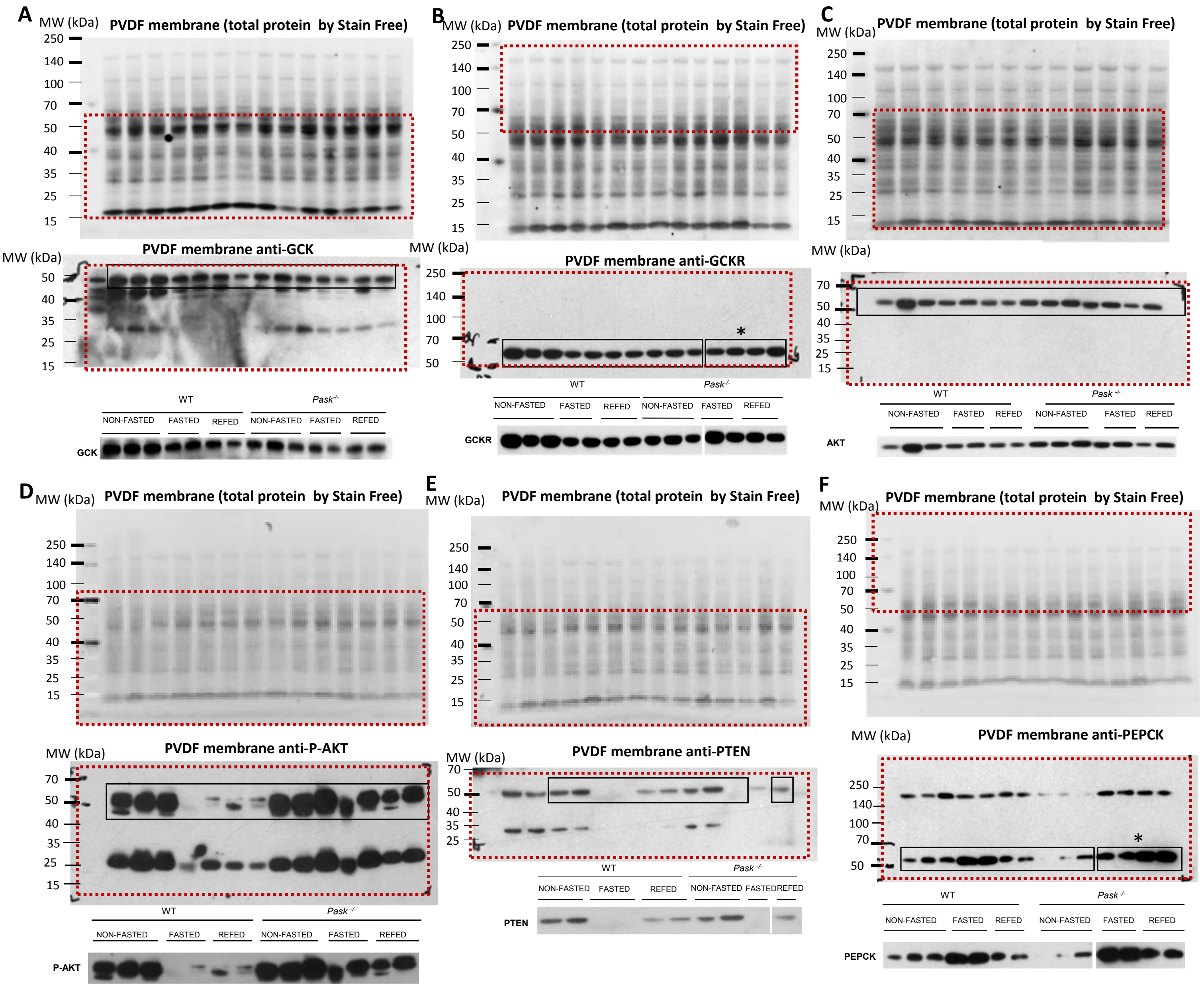

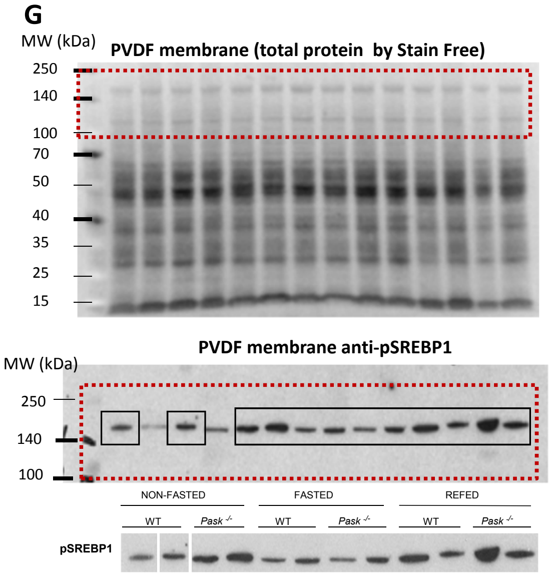

Supplementary Figure 1. Full-length stain free PVDF membranes and blot images showed in the main figures. The PVDF membranes were cropped (dotted red line) in order to save the use of large amounts of expensive antibodies, and then incubated with each specific antibody. The regions of membrane incubated, were according to the molecular weigh of protein to be detected. The boxed bands correspond to the cropped blots that appear in the main figure. The fragment labeled with * was horizontally flipped in the cropped final figure, in order to be consistent with the normal arrangement indicated in the rest of the graphs (firstly fasted and then refed). These conditions were loaded inverted in the gel. Final cropped blots are displayed as appear in the main figures. **(A)** GCK, **(B)** GCKR, **(C)** AKT, **(D)** P-AKT, **(E)** PTEN, , **(F)** PEPCK, **(G)** pSREBP1
